# A Law to Regulate the Practice of Dentistry in the State of Ohio

**Published:** 1868-04

**Authors:** 


### A LAW TO REGULATE THE PRACTICE OF DENTISTRY IN THE STATE OF OHIO.

Section 1. *Be it enacted by the General Assembly of the State of Ohio*, That it shall be unlawful for any person to practice Dentistry in the State of Ohio for compensation, unless such person has received a Diploma from the faculty of a Dental College duly incorporated under the laws of this or any other State of the United States, or foreign country, or a certificate of qualification issued by the State Dental Society, or by any local Society auxiliary thereto; provided, that nothing in this section shall apply to persons now engaged in the practice of Dentistry in this State, before the first day of January, 1873.

SEC. 2. Any person who shall practice Dentistry without having complied with the regulations of this act, shall be deemed guilty of a misdemeanor, and upon conviction thereof, shall be fined not less than fifty dollars nor more than two hundred dollars; provided, that nothing in this act shall be construed to prevent physicians and surgeons from extracting teeth.

SEC. 3. All prosecutions under this act shall be by indictment before the Court of Common Pleas in the County where the offense was committed, and all fines imposed and collected under the provisions of this act, shall be paid into the Treasury of the County where such conviction shall take place, for the use of the Common Schools within such County.

SEC. 4. This act shall take effect and be in force from and after its passage.

